# Variation in *HNF1B* and Obesity May Influence Prostate Cancer Risk in African American Men: A Pilot Study

**DOI:** 10.1155/2013/384594

**Published:** 2013-12-09

**Authors:** Ganna Chornokur, Ernest K. Amankwah, Stacy N. Davis, Catherine M. Phelan, Jong Y. Park, Julio Pow-Sang, Nagi B. Kumar

**Affiliations:** ^1^Department of Cancer Epidemiology, Moffitt Cancer Center, Tampa, FL 33612, USA; ^2^The Center for Equal Health, Tampa, FL 33612, USA; ^3^Department of Health Outcomes and Behavior, Moffitt Cancer Center, Tampa, FL 33612, USA; ^4^Department of Genitourinary Oncology, Moffitt Cancer Center, Tampa, FL 33612, USA

## Abstract

*Background*. Prostate cancer (PCa) racial disparity is multifactorial, involving biological, sociocultural, and lifestyle determinants. We investigated the association between selected potentially functional polymorphisms (SNPs) and prostate cancer (PCa) risk in Black (AAM) and White (EAM) men. We further explored if these associations varied by the body mass index (BMI) and height. *Methods*. Age-matched DNA samples from 259 AAM and 269 EAM were genotyped for 10 candidate SNPs in 7 genes using the TaqMan allelic differentiation analysis. The dominant, recessive, and additive age-adjusted unconditional logistic regression models were fitted. *Results*. Three SNPs showed statistically significant associations with PCa risk: in AAM, *HNF1B *rs7501939 (OR = 2.42, *P* = 0.0046) and rs4430796 (OR = 0.57, *P* = 0.0383); in EAM, *CTBP2* rs4962416 (OR = 1.52, *P* = 0.0384). In addition, high BMI in AAM (OR = 1.06, *P* = 0.022) and height in EAM (OR = 0.92, *P* = 0.0434) showed significant associations. Interestingly, *HNF1B* rs7501939 was associated with PCa exclusively in obese AAM (OR = 2.14, *P* = 0.0103). *Conclusion*. Our results suggest that variation in the *HNF1B* may influence PCa risk in obese AAM.

## 1. Introduction

Prostate cancer (PCa) remains the most common type of solid malignancy and the second-leading cause of all cancer death in US men [1]. However, the burden of PCa is not the same across all racial and ethnic groups, as African American/Black men (AAM) consistently demonstrate 1.6 times higher incidence and 2-3 times higher mortality rates of PCa, compared to their nonhispanic white (EAM) counterparts [2]. In addition, AAM are more likely to be diagnosed at an earlier age and have more aggressive tumors and higher recurrence rates following definite treatments [2, 3]. The etiology of racial disparity in PCa is thought to be multifactorial, involving biological, sociocultural, and lifestyle determinants [4]. Although genome-wide association studies (GWAS) have identified more than a dozen PCa risk loci [5], elucidating the biological basis for these associations is challenging [6]. Identified risk loci include the noncoding variants, such as those located in the 8q24 region [7], as well as polymorphisms in the coding regions (genes) that either alter, or are predicted to alter, the protein expression (such as HNF1B [8], TERT [5], and RNASEL [9]). The post-GWAS studies are increasingly suggestive of the interaction between genetic variants and environmental risk factors [10] for which our understanding is still largely inadequate [11].

Established risk factors for PCa are increasing age, race, and family history of the disease [2–4]. Obesity (which affects 35% of all US adults [12] and is more prevalent in African American population [13]) is linked to a plethora of diseases including cardiovascular problems, type II diabetes, gallbladder disease, and osteoarthritis [14], and an array of human cancers such as breast, uterine, and pancreas [15, 16]. Furthermore, obesity alters the individual's biochemical and hormonal profile [17], which may facilitate cancer growth [18]. However, obesity has been inconsistently associated with PCa risk [19], and the inconsistency may be due to an interaction with genetic variants [20, 21]. In an attempt to elucidate the connection between PCa health disparity, genetic variation, and obesity, we hypothesized that genetic variation differentially alters the PCa risk in obese and nonobese AAM and EAM. Given the extremely high burden of PCa and staggering rates of obesity, elucidating the links between the individual's genetic variation, race, PCa risk, and obesity is likely to have a major positive impact on the public health of the US population. Thus, our hypothesis-generating study may open new venues for tackling the PCa disparity from a new perspective.

## 2. Methods

### 2.1. Study Participants

Study participants were recruited from various clinics in the Tampa Bay area in Florida, including the Moffitt Cancer Center, Tampa Bay Radiation Oncology centers, Moffitt Cancer Center affiliated-Lifetime Cancer Screening & Prevention Center, James A. Haley Veteran Affairs (VA), and the 30th Street Medical Associates (a community clinic). All recruitment protocols were approved by the University of South Florida Institutional Review Board (IRB), while the VA protocol was approved by the VA IRB. The study population comprised of AAM and EAM aged 30–85 years and enrolled between 2006 and 2012. The cases and controls were recruited during the initial PCa screening of all consecutive, unselected patients. Cases were histologically confirmed PCa patients and controls were men with low PSA and/or no evidence of PCa on biopsy. The AAM or EAM ancestry was self-reported. Men were excluded if they did not self-identify as either AAM or EAM, were outside of the 30–85 year old range, were in poor physical or mental health, were diagnosed with other cancers (excluding nonmelanoma skin cancer), or did not speak English well enough to read and understand the informed consent. The response rates in all studies were high, at or above 90%.

### 2.2. Single Nucleotide Polymorphism (SNP) Selection and Genotyping

Literature search using PubMed and Google scholar databases was performed to identify potentialSNPs of interest. The following criteria were set to guide the SNP search (all inclusive): (1) confer increased PCa risk in AAM; (2) confer increased PCa risk in EAM; (3) demonstrate potential for functional significance (i.e., located in or close to a gene with a known function); (4) reported minor allele frequency (MAF) ≥ 15% in AAM and EAM. Based on these criteria, 10 SNPs in 7 genes were selected: rs4430796; rs7501939; rs1859962 in *HNF1B*; rs10993994 in *MSMB*; rs822396 in *ADIPOQ*; rs4263970; rs4612601 in *EPHB2*; rs4962416 in *CTBP2*; rs627839 in *RNASEL*; rs2070874 in *IL4*. All these SNPs have reported functional significance (actual or hypothesized). DNA was extracted from blood or buccal cell samples using commercially available extraction kits and TaqMan genotyping was conducted at the Moffitt Cancer Center on the DNA samples from 259 (136 cases and 123 controls) AAM and 269 (147 cases and 142 controls) EAM, matched on age at diagnosis.

### 2.3. Statistical Analyses

Descriptive statistics were used to summarize participants' demographic and clinical characteristics. Genotypes among participants were used to estimate allele frequencies and departure from Hardy-Weinberg equilibrium (HWE) was assessed among control subjects using Chi-squared test. The association between each SNP and PCa risk was estimated with odds ratios (OR) and 95% confidence intervals (CI) using unconditional logistic regression adjusted for age at diagnosis. Three inheritance genetic models (log-additive, dominant, and recessive) were tested for each SNP and the model with the minimum *P* value was considered as the best fitting model. Separate analyses were performed for each race and all men combined. We conducted exploratory subgroup analyses for different strata based on BMI and height within each race. Statistical tests were two sided with an alpha level <0.05 considered statistically significant. All statistical analyses were performed with SAS/Genetics version 9.2 (SAS Institute, NC, USA).

## 3. Results

Selected characteristics of the study participants by case/control status are shown in Table 1. For both ethnic groups, men were likely to be between 50 to 64 years of age and between 68 and 72 inches tall. AAM were more likely to be obese than EAM (47% and 35.5% obese men in each group, resp.). AAM were also more likely to be taller. Within each ethnic group, cases were more likely to be older, although there was no significant difference in the age of cases and controls between races. In addition, AAM cases were more likely to be obese than AAM controls. In contrast, EAM controls were more likely to be obese than EAM cases.

The results for association analyses between SNP and risk are shown in Table 2. The MAF of the SNPs ranged from 0.15–0.49 and none of the SNPs deviated from HWE (all *P* > 0.05). In AAM, we observed an increased PCa risk at *HNF1B* rs7501939 (recessive model: OR = 2.42, 95%  CI = 1.31–4.47, *P* = 0.0046; additive model: OR = 1.56, 95% CI = 1.08–2.27, *P* = 0.0193) and a decreased PCa risk at *HNF1B* rs4430796 (dominant model: OR = 0.57, 95% CI = 0.34–0.97, *P* = 0.0383; additive model: OR = 0.67, 95% CI = 0.46–0.99, *P* = 0.0431). These SNPs were not significantly associated with PCa risk in EAM. In EAM, we observed an increased PCa risk at the *CTBP2* rs4962416 (dominant model: OR = 1.69, 95%  CI = 1.02–2.80, *P* = 0.0415; additive model: OR = 1.52, 95%  CI = 1.02–2.26, *P* = 0.0384). This association was not confirmed in AAM. We attempted to analyze for the interaction between SNPs and race in multivariable model. None of SNP and race interaction is significant (*P* > 0.05).

Age-adjusted association between selected anthropometric variables and PCa are shown in Table 3. We observed an increased PCa risk in obese, compared to nonobese AAM (OR = 1.06, 95% CI = 1.01–1.11, *P* = 0.022) and decreased PCa risk in the tallest group of EAM compared to all other EAM (OR = 0.92, CI = 0.85–0.99, *P* = 0.0434). Since our results indicated that BMI might be positively associated with PCa in AAM, we decided to examine the SNP-associations stratified by obesity for SNPs that were significant for AAM: rs7501939 and rs4430796.

Results for SNP-associations stratified by obesity in AAM are shown in Table 4. Interestingly, when stratified by obesity status, rs7501939 at *HNF1B* only increased PCa risk in obese AAM (OR = 2.14, 95%CI = 1.2–3.8, *P* = 0.01) but not in the nonobese AAM (*P* = 0.76) or EAM of any BMI (*P* = 0.3 in obese, and *P* = 0.8 in nonobese EAM). No differential association with obesity status was observed at rs4430796 in AAM (*P* = 0.18 in obese and *P* = 0.51 in the nonobese AAM). There were no significant associations with PCa risk at these SNP in EAM regardless of obesity status (*P* > 0.05).

Finally, we used the SNP Annotation and Proxy Search (SNAP) software (Broad Institute) to elucidate whether there are other SNPs in linkage disequilibrium (LD) with our significant SNPs. The results are shown in Table 5. There are no SNPs in the LD (*r* ^2^ ≥ 0.8) with either of the *HNF1B* SNPs significant in AAM. While there are several SNPs in weak LD (*r* ^2^ ≥ 0.5) with rs7501939, they were not found to be associated with PCa or any other disease. In EAM, while a number of SNPs are reported to be in LD with the CTBP2 rs4962416, only one of those SNPs (rs12769019) was linked to a marginally increased PCa risk in EAM (OR = 1.1, 95% CI, 0.99–1.25) [22].

## 4. Discussion

We observe that the *HNF1B* SNPs (rs7501939 and rs4430796) identified in PCa GWAS [23, 24] are associated with PCa risk in AAM and that the *CTBP2* SNP rs4962416, also identified in PCa GWAS [23], is associated with PCa risk in EAM. Our novel finding is that the association of rs7501939 with PCa risk in AAM may be modified by obesity.

In this study we observed that the *HNF1B* SNPs (rs7501939 and rs4430796) identified in PCa GWAS [23, 24] are associated with PCa risk in AAM and that the *CTBP2* SNP (rs4962416), also identified in PCa GWAS [23], are associated with PCa risk in EAM. Our novel finding is that the association of rs7501939 with PCa risk in AAM may be modified by obesity.

Gudmundsson et al. [24] reported mixed results regarding the association of PCa risk and *HNF1B* SNPs in EAM (largely living in Europe). Similar to our findings, *HNF1B* SNP rs4430796 was not significantly associated with PCa in the EAM; however,* HNF1B* SNP rs7501939 was significantly associated with PCa in the EAM in their sample. The authors also reported that the same SNPs were significantly inversely associated with type II diabetes. Stevens et al. [8] reported that *HNF1B* SNPs (rs7501939 and rs4430796) were both significantly inversely associated with PCa risk in their cohorts of EAM. However, unlike Gudmundsson et al. [24], the authors did not observe a statistically significant association with diabetes. Liu et al. [25] conducted a meta-analysis of all PCa-related GWA studies. The authors have found that in combined analysis, *HNF1B* SNPs (rs7501939 and rs4430796) were significantly associated with PCa. However, when stratified by ethnicity, neither of the two SNPs attained statistical significance when analysis was restricted to AAM. Sun et al. [26] analyzed a diverse cohort of men (2139 EAM and 717 AAM) and reported significant associations with PCa risk and both SNPs in EAM. However, when analysis was restricted to AAM, only SNP rs4430796 retained statistical significance. This may be due to Sun et al. who used major alleles as risk alleles (T for rs4430796 and G for rs75001939), while our study and other cited studies used minor alleles as risk alleles. Ahn et al. [27] reported that SNP rs4430796 was not associated with risk for metastatic prostate cancer and recurrence.

Studies that investigated PCA risk and *HNF1B* SNPs within large AAM cohorts also found similar mixed results. Chang et al. [28] could not validate the association of PCa risk and *HNF1B* SNPs (rs7501939 and rs4430796) in their large cohort of 4,040 AAM PCa cases and 3,748 healthy AAM controls. Haiman et al. [29] also reported no association in their large cohort of 3,621 AAM PCa cases and 3,652 AAM controls. Hooker et al. [30] found mixed results in their attempts to replicate the PCa SNPs identified in the GWAS studies. In a cohort of 755 unrelated AAM, *HNF1B* rs7501939 was not associated with PCa risk, while HNF1B rs4430796 conferred significant increased PCa risk.

None of the aforementioned studies controlled for BMI in the association between PCa risk and *HNF1B* SNPs (rs7501939 and rs4430796). However, Lindstrom et al. [31] investigated the modifying effects of BMI on PCA risk and different SNPs in EAM. Lindstrom et al. found that BMI does not have modifying effects on the SNP-PCa associations in EAM; similar analysis was not performed in AAM.

As can be concluded from the referenced studies, there is significant discordance between the published studies on the effects of the two aforementioned *HNF1B* SNPs on PCa risk. While the authors presented high-quality studies with large sample sizes, the majority did not report controlling for the environmental confounders, including BMI. To our knowledge, we are the first group to report that the association of the *HNF1B* SNPs with PCa risk may be modified by the level of adiposity and racial/ethnic background. Importantly, BMI was associated with PCa risk in our sample of AAM independently of genetic variation (OR = 1.06, *P* = 0.022) (Table 3). Our hypothesis-generating data may be even more intriguing in light of the ongoing debate surrounding the relationship between obesity and PCa risk. At present, there appears to be a consensus that obese men experience slightly reduced overall PCa incidence, at the expense of an increase in aggressive, “clinically significant” disease. The overall decreased PCa risk, therefore, is mainly due to reduction in the “clinically insignificant,” potentially indolent PCa [17]. While several hypotheses aimed to explain that this observation have been proposed [32], neither has been widely accepted by scientific or medical communities. Additionally, because the data were obtained in EAM, the effect of obesity on PCa risk in AAM remains unknown. Our pilot study adds information to close this knowledge gap and serve as a basis for future sufficiently powered studies involving larger numbers of AA participants, as well as more extensive epidemiological data analysis.

Mutations in the *HNF1B* cause MODY5 (maturity-onset of diabetes, type 5) that may be accompanied by urinary tract disorders, including renal disease and/or undeveloped/malformed kidneys and atrophic pancreas [33, 34]. Some men with the *HNF1B* mutations have malformations in the reproductive tract including epididymal cysts, agenesis of the vas deferens, or infertility due to abnormal spermatozoa [35]. More recently, genetic variants in the *HNF1B* were implicated in the prostate [36, 37] and endometrial [38, 39] cancer risks. It was reported [40] that different *HNF1B* isoforms were expressed in prostate tumors versus normal prostate tissue, thus providing functional evidence for a potential role of this gene in PCa. However, the functional studies to examine whether *HNF1B* variants influence PCa risk and/or prognosis are lacking. *HNF1B* variants were implicated in a slightly reduced risk of type II diabetes mellitus in AAM and EAM [24]. Interestingly, PCa risk seems to be attenuated in men with diabetes [41], although the latter was only reported in people of EA descent and the biological basis for this association remains to be elucidated. At this time, the relationship between obesity, diabetes, and PCa is poorly understood, and so is the contribution of the *HNF1B* variants to the risk. However, since obesity as a risk factor for breast cancer differs in AA and EA women [42, 43], it is plausible that a similar effect may be observed with obesity and PCa in AAM and EAM. Additionally, our data suggests that *HNF1B *variants may be implicated in the risk. Future epidemiological, genetic, and functional tumor biology studies are required to address this provocative hypothesis.

In EAM, our results replicate a previously reported finding that the *CTBP2* rs4962416 confers increased PCa risk: OR = 1.69, *P* = 0.0415 in our study versus the overall GWAS data: OR = 1.25, *P* = 0.004 [25]. *CTBP2* encodes a transcriptional corepressor that is activated under stress condition and can mediate stress-induced migration of tumor cells [44]. *CTBP2* expression is detected in the prostate and has been linked to decreased *PTEN* expression and activation of the phosphatidylinositol 3-kinase pathway [45] which may support or promote PCa growth. rs4962416 was not previously implicated in PCa risk in AAM [25] which is also in agreement with our data.

The main strength of our study is inclusion of equal proportions of age-matched AA and EA men with high rates of obese men in both populations, allowing us to tease out the interaction of obesity with genetic variants. Our results should be interpreted in light of limitations of a small sample size and inability to access relevant information such as smoking/drinking behavior and diabetes history. However, given the increased PCa burden in AAM, alarming obesity rates, and limited number of studies that involve AAM, our results are novel, timely, and as such, deserve dissemination.

In summary, our results suggest that germline genetic variation in *HNF1B* and *CTBP2* differentially contribute to PCa risk in men of different races and adiposities. Future sufficiently powered studies involving a larger proportion of AAM are needed to elucidate the potential connection between the race, PCa, obesity, diabetes, and *HNF1B*. In addition, functional tumor biology studies are warranted to elucidate the effects of obesity on PCa in carriers and noncarriers of different races and ethnicities.

## Figures and Tables

**Table 1:** Selected characteristics of the study participants by case/control status.

	AAM: 259 total		EAM: 269 total		Total sample combined	
	No. of cases (%) 136 (52.5)	No. of controls (%) 123 (47.5)	No. of cases (%) 147 (54.6)	No. of controls (%) 122 (45.4)	No. of cases (%) 283 (53.6)	No. of controls (%) 245 (46.4)
Age						
<50	11 (8)	12 (8)	19 (16)	30 (24)	30 (12)	42 (16)
50–64	74 (54)	80 (54)	85 (70)	73 (59)	159 (62)	153 (56.5)
≥65	51 (38)	55 (38)	18 (14)	17 (14)	69 (26)	72 (26)
Missing	NA	NA	NA	3 (3)	NA	3 (1.5)

BMI						
≤24.9 normal weight	9 (7)	25 (17)	28 (23)	26 (21)	37 (15)	51 (19)
25.0–29.9 overweight	47 (34.5)	70 (48)	47 (38)	46 (37)	94 (36)	116 (42.5)
≥30.0 obese	71 (52)	48 (33)	46 (38)	51 (42)	58.5 (45)	49.5 (37.5)
Missing	9 (6.5)	4 (2)	1 (1)	NA	10 (3.5)	4 (1)

Height						
≤67	30 (22)	26 (17.5)	22 (18)	21 (17)	52 (20)	47 (17)
68–72	65 (48)	98 (66.5)	79 (65)	74 (60)	144 (56.5)	172 (63)
≥73	32 (23.5)	19 (13)	21 (17)	28 (23)	53 (20)	47 (18)
Missing	9 (6.5)	4 (3)	NA	NA	9 (3)	4 (1.5)

**Table 2:** Age-adjusted odds ratios (OR) and 95% confidence intervals (95% CI) for PCa in AAM and EAM and combined.

Rs# and gene; minor allele and its frequency	Dominant model OR (CI); *P* value	Recessive model OR (CI); *P* value	Additive model OR (CI); *P* value
rs4612601; EPHB2 AA: 0.49 (G) EA: 0.45 (A)	AAM: 1.09 (0.63–1.9); 0.749	0.86 (0.46–1.61); 0.638	0.99 (0.69–1.41); 0.951
	EAM: 1.18 (0.67–2.10); 0.564	0.9 (0.52–1.56); 0.709	1.02 (0.73–1.43); 0.912
	All: 1.13 (0.76–1.69); 0.537	0.89 (0.59–1.34); 0.565	1.00 (0.79–1.28); 0.969

rs4263970; EPHB2 AA: 0.47 (T) EA: 0.45 (C)	AAM: 0.98 (0.57–1.68); 0.932	0.88 (0.44–1.75); 0.711	0.95 (0.66–1.38); 0.797
	EAM: 1.08 (0.61–1.9); 0.795	0.76 (0.44–1.33); 0.334	0.93 (0.66–1.30); 0.665
	All: 1.02 (0.69–1.5); 0.918	0.81 (0.53–1.25); 0.343	0.94 (0.73–1.21); 0.630

rs822396; ADIPOQ AA: 0.2 (G) EA: 0.15 (G)	AAM: 0.89 (0.52–1.52); 0.671	1.64 (0.37–7.21); 0.511	0.96 (0.6–1.54); 0.878
	EAM: 0.75 (0.44–1.27); 0.288	1.1 (0.29–4.09); 0.094	0.82 (0.53–1.29); 0.392
	All: 0.82 (0.56–1.19); 0.285	1.29 (0.49–3.48); 0.601	0.88 (0.64–1.22); 0.456

rs10993994; MSMB AA: 0.2 (C) EA: 0.34 (T)	AAM: 0.82 (0.48–1.4); 0.472	1.04 (0.51–2.10); 0.919	0.92 (0.63–1.33); 0.657
	EAM: 1.04 (0.54–2.00); 0.899	0.61 (0.36–1.05); 0.077	0.81 (0.56–1.17); 0.258
	All: 0.9 (0.6–1.35); 0.611	0.75 (0.49–1.14); 0.177	0.87 (0.67–1.11); 0.261

rs1859962; HNF1B AA: 0.49 (G) EA: 0.2 (G)	AAM: 1.03 (0.61–1.74); 0.905	1.23 (0.54–2.81); 0.626	1.06 (0.72–1.56); 0.752
	EAM: 1.38 (0.77–2.45); 0.278	1.00 (0.57–1.76); 0.997	1.13 (0.79–1.6); 0.508
	All: 1.16 (0.8–1.69); 0.431	1.06 (0.67–1.69); 0.790	1.09 (0.85–1.4); 0.503

rs7501939; HNF1B AA: 0.48 (C) EA: 0.49 (T)	AAM: 1.34 (0.73–2.47); 0.343	**2.42 (1.31–4.47); 0.0046**	**1.56 (1.08–2.27); 0.0193**
	EAM: 0.82 (0.5–1.35); 0.439	1.13 (0.54–2.38); 0.742	0.93 (0.65–1.33); 0.692
	All: 0.99 (0.68–1.45); 0.981	**1.76 (1.11–2.79); 0.0167**	1.18 (0.92–1.52); 0.187

rs4430796; HNF1B AA: 0.34 (A) EA: 0.47 (A)	AAM: **0.57 (0.34–0.97); 0.0383**	0.64 (0.29–1.42); 0.272	**0.67 (0.46–0.99); 0.0431**
	EAM: 1.27 (0.7–2.3); 0.439	0.98 (0.57–1.68); 0.946	1.07 (0.76–1.51); 0.689
	All: 0.81 (0.55–1.18); 0.273	0.86 (0.59–1.33); 0.500	0.87 (0.69–1.12); 0.279

rs2070874; IL4 AA: 0.45 (T) EA: 0.2 (T)	AAM: 0.94 (0.54–1.64); 0.824	1.45 (0.7–3.0); 0.314	1.08 (0.73–1.59); 0.700
	EAM: 0.65 (0.38–1.11); 0.115	0.92 (0.22–3.93); 0.916	0.72 (0.45–1.14); 0.161
	All: 0.82 (0.57–1.17); 0.275	1.32 (0.7–2.49); 0.386	0.94 (0.71–1.24); 0.649

rs627839; RNASEL AA: 0.46 (T) EA: 0.45 (T)	AAM: 1.2 (0.7–2.06); 0.511	1.63 (0.78–3.42); 0.192	1.24 (0.85–1.83); 0.252
	EAM: 0.86 (0.5–1.47); 0.571	0.99 (0.55–1.8); 0.986	0.94 (0.67–1.32); 0.710
	All: 1.01 (0.69–1.48); 0.952	1.21 (0.76–1.91); 0.427	1.06 (0.83–1.37); 0.632

rs4962416; CTBP2 AA: 0.23 (C) EA: 0.23 (C)	AAM: 0.9 (0.5–1.59); 0.709	5.65 (0.62–51.7); 0.125	1.05 (0.63–1.74); 0.861
	**EAM: 1.69 (1.02–2.8); 0.0415**	1.72 (0.68–4.31); 0.251	**1.52 (1.02–2.26); 0.0384**
	All: 1.28 (0.88–1.85); 0.198	2.12 (0.92–4.9); 0.0784	1.31 (0.97–1.78); 0.083

Bolded values denote statistically significant associations.

**Table 3:** Age-adjusted association between selected anthropometric variables and PCa in men stratifying by race and combined.

	AAM	EAM
	OR (95% CI); *P* value	OR (95% CI); *P* value
Height (inches)	0.98 (0.9–1.05); 0.54	**0.92 (0.85–0.99); 0.0434**
BMI (kg/m^2^)	**1.06 (1.01–1.11); 0.022**	0.98 (0.93–1.023); 0.33
<30 (nonobese)	1.0	1.0
≥30 (obese)	**2.07 (1.21–3.55); 0.008**	0.90 (0.54–1.51); 0.69

Bold denotes statistically significant associations.

**Table 4:** rs7501939 and rs4430796 as PCa risk factors in AAM stratified by BMI.

	Nonobese AAM	Obese AAM	All AAM
rs7501939	1.09 (0.65–1.83); 0.75	**2.13 (1.20–3.81); 0.01**	**1.56 (1.08–2.27); *P* = 0.019**
rs4430796	0.84 (0.49–1.43); 0.52	0.67 (0.37–1.20); 0.18	**0.67 (0.46–0.99); 0.043**

Statistically significant associations are shown in bold. Data is age adjusted.

**Table 5:** SNPs reported to be in LD regions with our SNPs of interest.

SNP of interest	*r* ^2^ ≥ 0.8	*r* ^2^ ≥ 0.5	Disease and comments
rs7501939 in AAM	None	rs11657964 (*r* ^2^ = 0.61) rs8064454 (*r* ^2^ = 0.549)	None reported to be associated with prostate cancer or any other disease

rs4430796 in AAM	None	None	NA

rs4962416 in EAM	rs4962720 (*r* ^2^ = 1.00) rs4962419 (*r* ^2^ = 1.00) rs12771627 (*r* ^2^ = 1.00) rs12769019 (*r* ^2^ = 0.92) rs11598549 (*r* ^2^ = 0.92) rs12782469 (*r* ^2^ = 0.83)	14 additional SNPs	rs12769019: slightly increased risk for prostate cancer in EAM (OR = 1.1) [19]
